# Amorphous nanoparticles in clays, soils and marine sediments analyzed with a small angle X-ray scattering (SAXS) method

**DOI:** 10.1038/s41598-021-86573-9

**Published:** 2021-03-26

**Authors:** Katsuhiro Tsukimura, Youko Miyoshi, Tetsuich Takagi, Masaya Suzuki, Shin-ichiro Wada

**Affiliations:** 1 Institute for Geo-Resources and Environment, AIST (National Institute of Advanced Industrial Science and Technology), Tsukuba, Ibaraki 305-8567 Japan; 2 Faculty of Agriculture, Kyusyu University, Fukuoka, Fukuoka 819-0395 Japan

**Keywords:** Mineralogy, Nanoparticles

## Abstract

This paper describes the amounts and size distributions of amorphous nanoparticles in clays, soils and marine sediments, and the effect of amorphous nanoparticles on the properties of clays, soils and marine sediments. So far aluminum–silicate amorphous nanoparticles such as allophane were observed only in soils of volcanic origin with a transmission electron microscope, and thus most people believed that aluminum–silicate amorphous nanoparticles were present only in soils of special origin. Recently, a method has been devised to quantify amorphous nanoparticles by using small angle X-ray scattering intensity. Using the method, we have quantified amorphous nanoparticles in clays, soils and marine sediments, and have found that all clays, soils and marine sediments measured in this study contain large amounts of amorphous nanoparticles. On the basis of this result, we have concluded that large amounts of amorphous nanoparticles are ubiquitously formed from rocks when the rocks are weathered or altered. We have also found that the amorphous nanoparticles affect the properties of clays, such as adsorption properties and plasticity. These findings show that amorphous nanoparticles play an important role in clays, soils and marine sediments.

## Introduction

Amorphous nanoparticles such as allophane (aluminum–silicate)^1–6^ and ferrihydrite (iron-oxyhydroxide)^7^ were observed only in a limited variety of sample types with a transmission electron microscope (TEM). Allophane was observed only in soils of volcanic origin, and ferrihydrite only in iron-rich samples taken from sediments, soils, mine-wastes and mine-drainage^8^. These results have led us to believe that amorphous nanoparticles are present only in limited samples of special origin. Therefore, most people usually do not try to observe amorphous nanoparticles in clays, soils and marine sediments with TEM. Unless one tries to observe amorphous nanoparticles with TEM, one cannot observe amorphous nanoparticles with TEM even if the sample contains amorphous nanoparticles. This is partly because finding amorphous nanoparticles in the sample with TEM is difficult compared with crystalline minerals; amorphous nanoparticles have small and indistinct shapes, while crystalline minerals have large and distinct shapes. As a result, TEM may have overlooked the amorphous nanoparticles in samples.

**Figure 1:**
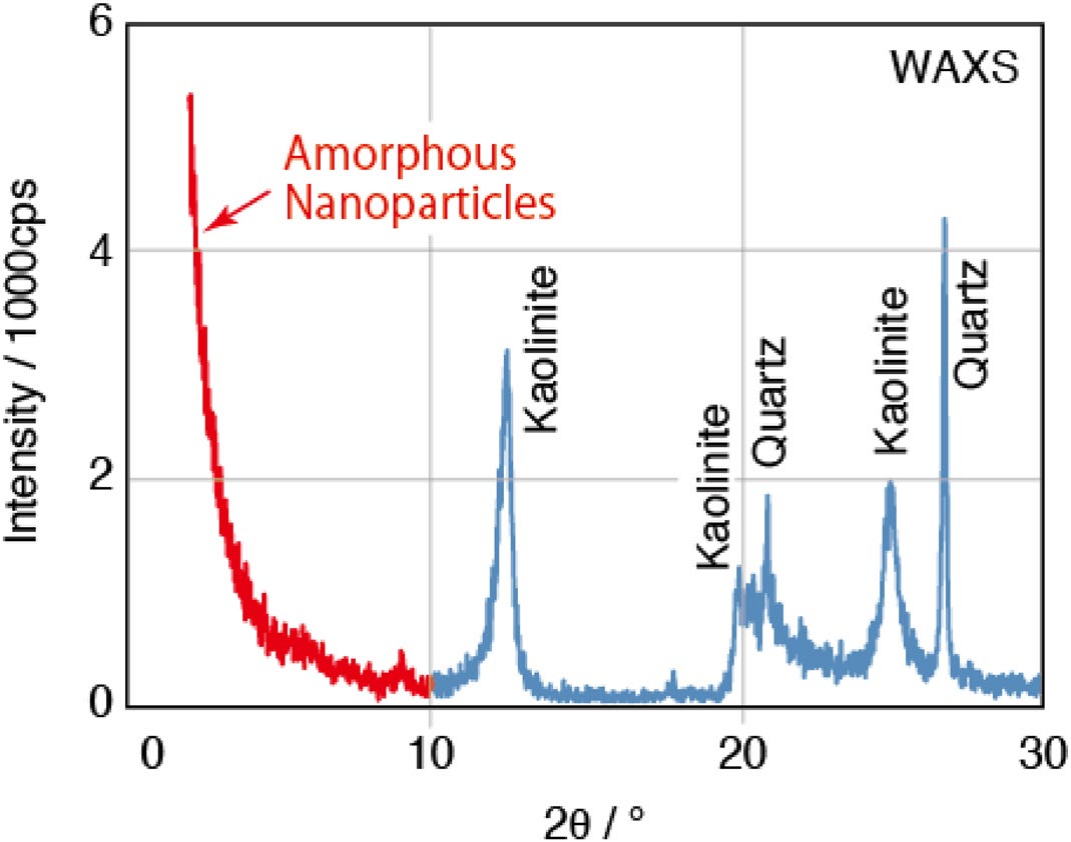
Wide angle X-ray scattering (WAXS) for Kibushi Kaolin, Aichi, Japan. Scattering depicted in red are from amorphous nanoparticles.

On the other hand, X-ray scattering method will be the promising one for analyzing amorphous nanoparticles in clays, soils and marine sediments. Figure 1 shows an X-ray scattering pattern of Kibushi Kaolin, Aichi, Japan^9^. In this scattering pattern we can see some reflections from kaolinite and quartz at 2θ > 10° (Cu *Kα*). Observing this scattering pattern, most people will describe only kaolinite and quartz and will not notice the presence of amorphous nanoparticles. This scattering pattern, however, includes small angle X-ray scattering (SAXS) from amorphous nanoparticles, which is background-like scattering depicted in red at 2θ < 10° (Cu* Kα*) (Fig. 1). The strong SAXS from amorphous nanoparticles are always present in the scattering patterns of clays, soils and marine sediments^9–15^, which shows that clays, soils and marine sediments always contain amorphous nanoparticles. By analyzing these SAXS intensities, we may be able to quantify amorphous nanoparticles in a geologic sample.

Recently a method has been devised for quantifying amorphous nanoparticles in a geologic sample using SAXS intensity^16^. Using the method, we have quantified amorphous nanoparticles in 29 clays, five soils, and two marine-sediments, and found that these samples contain large amounts of amorphous nanoparticles. On the basis of the SAXS intensities we have also calculated the size distribution of amorphous nanoparticles and the distance distribution between the centers of two amorphous nanoparticles. In this calculation, we use decoupling approximation^16,17^.

We can expect that amorphous nanoparticles in materials affect the properties of the materials, such as adsorption property and plasticity. Large amount of water and carbon dioxide is adsorbed by synthetic aluminum–silicate amorphous nanoparticles^18^. This is because the amorphous nanoparticles have the large specific surface area. In this study we have shown that amorphous nanoparticles have large specific surface areas, and that amorphous nanoparticles adsorb moderate amounts of methylene blue and large amounts of water. Plasticity of clays may increase with increasing amounts of amorphous nanoparticles. We can consider that small, near-spherical shapes of amorphous nanoparticles are advantageous for giving the plasticity to materials. In this study we have found that clays with high plasticity contain large amounts of amorphous nanoparticles.

## Results

### Samples

Table 1 describes 29 clays, five soils, and two marine sediments measured in this study. The 29 clays that we measured include nine reference samples from the Clay Science Society of Japan, 11 source clay samples from the Clay Minerals Society, and nine samples collected by authors. Each clay sample contains one kind of clay mineral. The reference clays from the Clay Science Society of Japan contain smectite (montmorillonite, or saponite), kaolinite, dickite, pyrophillite, illite, or vermiculite^19^. The source clays from the Clay Minerals Society contain smectite (beidellite, hectorite, montmorillonite, or nontronite), illite–smectite, illite, palygorskite, or sepiolite^20^. The clays collected by authors contain smectite (montmorillonite) or kaolinite^9,10^. The five soils collected by authors include one soil containing illite and four soils containing no clay mineral^2,5,21,22^. The two marine-sediments were collected by an author from volcanic deposits in Kagoshima Bay, Japan; one marine-sediment contains smectite (montmorillonite) and the other marine-sediment contains no clay mineral^11^. Table 2 shows the chemical compositions of all the samples. Supplementary Figure S1 shows wide angle X-ray scattering patterns for all the samples.

**Table 1:** Description of samples.

No	Source & symbol	Type	Clay mineral	Locality
571	JCSS-1101b	Clay	Kaolinite	Kanpaku, Tochigi, Japan
572	JCSS-1301	Clay	Dickite	Shokozan, Hiroshima, Japan
573	JCSS-2101	Clay	Pyrophillite	Shokozan, Hiroshima, Japan
574	JCSS-3101	Clay	Smectite (Montmorillonite)	Tsukinuno, Yamagata, Japan
575	JCSS-3102	Clay	Smectite (Montmorillonite)	Mikawa, Niigata, Japan
576	JCSS-5101	Clay	Illite	Nabeyama, Shimane, Japan
577	JCSS-5102	Clay	Illite	Nabeyama, Shimane, Japan
578	JCSS-3501	Clay	Smectite (Saponite)	Synthetic
579	KT Kanto loam	Soil	None	Setagaya, Tokyo, Japan
806	TT Gairome clay	Clay	Kaolinite	Seto, Aichi, Japan
807	TT Kibushi clay	Clay	Kaolinite	Seto, Aichi, Japan
808	TT Georgia-01	Clay	Kaolinite	Georgia, USA
809	TT Georgia-03	Clay	Kaolinite	Georgia, USA
861	CMS PFl-1	Clay	Palygorskite	Gadsden, Florida, USA
862	CMS SHCa-1	Clay	Smectite (Hectorite)	San Bernardino, California, USA
863	CMS STx-1	Clay	Smectite (Montmorillonite)	Gonzales, Texas, USA
864	CMS SWy-1	Clay	Smectite (Na-Montmorillonite)	Crook, Wyoming, USA
867	CMS SAz-1	Clay	Smectite (Montmorillonite)	Arizona, USA
868	CMS IMt-1	Clay	Illite	Montana, USA
869	CMS ISCz-1	Clay	Illite–smectite	Slovakia
870	CMS NAu-1	Clay	Smectite (Nontronite)	South Australia
871	CMS NAu-2	Clay	Smectite (Nontronite)	South Australia
872	CMS SepSp-1	Clay	Sepiolite	Valdemore, Spain
873	CMS SBId-1	Clay	Smectite (Beidellite)	Idaho, USA
874	TT Kawasaki	Clay	Smectite (Montmorillonite)	Kawasaki, Miyagi, Japan
875	TT Dobuyama	Clay	Smectite (Montmorillonite)	Dobuyama, Miyagi, Japan
876	JCSS-5501	Clay	Vermiculite	South Africa
877	YM PC-2	Sediment	Smectite (Montmorillonite)	Kagoshima Bay, Japan
878	YM PC-5	Sediment	None	Kagoshima Bay, Japan
879	YM Volclay	Clay	Smectite (Montmorillonite)	Wyoming, USA
901	TT Tsugaru-2	Clay	Smectite (Montmorillonite)	Tsugaru, Aomori, Japan
902	TT B-kou	Clay	Smectite (Montmorillonite)	Tsugaru, Aomori, Japan
903	SW W-235	Soil	None	Fukuoka, Japan
904	SW Togeyama	Soil	Illite	Fukuoka, Japan
905	SW W-155	Soil	None	Honolulu, Hawaii, USA
906	SW 1041	Soil	None	Choyo, Kumamoto, Japan

*JCSS* Japan clay science society, *KT* katsuhiro Tsukimura, *TT* Tetsuichi Takagi, *CMS* The clay mineral society, *YM* Youko Miyoshi, *SW* Shin-ichiro Wada.

**Table 2:** Chemical compositions of samples.

No	Chemical compositions/weight %												
	SiO_2_	TiO_2_	Al_2_O_3_	Fe_2_O_3_	FeO	MnO	MgO	CaO	Na_2_O	K_2_O	P_2_O_5_	H_2_O	CO_2_
571	43.9	0.1	36.6						0.1	0.8	0.2	18.3	
572	45.2	0.2	38.6						0.1	0.2	0.1	15.6	
573	67.9	0.2	23.3	0.2					0.1	0.1		8.5	
574	54.0	0.1	19.9	1.9			3.0	0.4	3.4	0.4		16.9	
575	66.4	0.1	11.9	1.6			2.6	0.5	2.0	1.3		13.6	
576	46.2	0.3	28.9	3.7			0.9	1.7	0.2	9.4	0.1	8.6	
577	45.4	0.2	28.3	4.0			1.0	2.0	0.1	9.2	0.1	9.7	
578	45.8		4.4				25.6	0.1	3.2	0.1		20.8	
579	31.3	2.1	27.8	19.1		0.3	2.1	0.6	0.3	0.6	0.2	18.9	
806	55.7	0.6	30.1	0.9			0.2	0.2	0.2	2.0		10.8	
807	49.5	0.9	32.5	0.9			0.2	0.3	0.1	0.8		15.4	
808	45.7	1.4	39.5	0.3			0.1	0.1	0.1	0.1		14.1	
809	46.5	1.6	38.4	0.4			0.1	0.1	0.1	0.2		13.9	
861	60.9	0.5	10.4	3.0	0.4	0.1	10.2	2.0	0.1	0.8	0.8	10.3	
862	34.7		0.7		0.3		15.3	23.4	1.3	0.1		21.6	
863	70.1	0.2	16.0	0.7	0.2		3.7	1.6	0.3	0.1		6.5	0.2
864	62.9	0.1	19.6	3.4	0.3		3.1	1.7	1.5	0.5		6.1	1.3
867	60.4	0.2	17.6	1.4	0.1	0.1	6.5	2.8	0.1	0.2		9.9	
868	49.3	0.6	24.3	7.3	0.6		2. 6	0.4		7.8		8.0	
869	51.6		25.6	1.1			2.5	0.7	0.3	5.4		10.2	
870	53.3		10.2	34.2			0.3	3.5	0.1				
871	57.0		3.4	37.4			0.3	2. 7	0.1				
872	52.9		2.6	1.2	0.3	0.1	23.6			0.1		20.8	
873	57.9	0.8	30.2	2.1			0.8	0.8	0.3	2.4		4.8	
874	72.8	0.1	10.4	1.4			2.7	0.9	1.1	0.2		4.6	5.7
875	58.3	0.2	12.6	3.0			4.9	1.9		0.1		8.3	10.7
876	39.9	1.0	8.8	8.0		0.1	23.5	4.5	0.1	4.9	1.6	7.2	
877	66.0	0.5	13.9	5.0		0.2	1.8	2.1	5.6	2.0	0.1	3.0	
878	54.5	0.4	11.9	3.5			20.0	0.4	4.1	0.8		4.4	
879	61.6	0.2	19.9	4.0			2.2	1.3	2.2	0.6	0.1	5.7	
901	62.7	0.4	15.4	3.4			4.6	0.9	1.1	0.6		11.0	
902	70.9	0.4	13.2	3.1		0.1	2.4	2.1	2.1	0.8	0.1	4.9	
903	69.7	1.3	12.7	4.6		0.1	1.0	1.1	0.9	1.6	0.2	7.9	
904	43.8	1.3	34.6	7.2		0.2	0.3	0.4	0.2	0.2		13.1	
905	27.0	3.8	23.3	33.8		0.5	0.5	0.1		0.5	0.1	11.7	
906	39.4	1.3	22.5	13.5		0.2	3.7	5.1	1.3	0.8	0.4	14.1	

### Weight % and average external radius of amorphous nanoparticles

**Figure 2:**
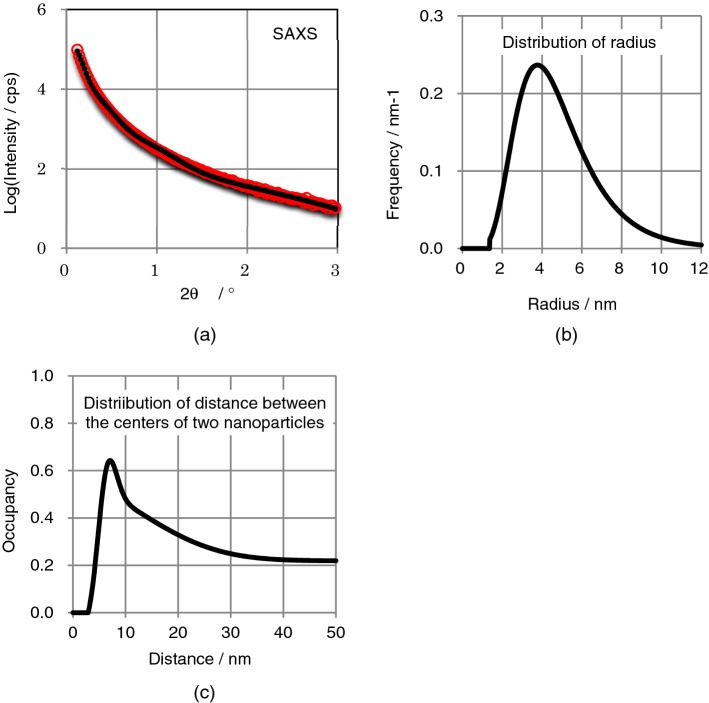
Small angle X-ray scattering (SAXS) data and their analytical results. (**a**) SAXS intensities. Red circles are observation and black dots are calculation. (**b**) Distribution of external radius of amorphous nanoparticles derived from SAXS intensities. (**c**) Distribution of distance between the centers of two amorphous nanoparticles derived from SAXS intensities.

Figure 2a shows small angle X-ray scattering (SAXS) pattern of Kibushi Kaolin, Aichi, Japan. By integrating the SAXS intensities over 3-dimensional reciprocal space, we have calculated the weight % of amorphous nanoparticles. By fitting the calculated SAXS intensities with the observed SAXS intensities, we have derived the distribution of external radius of amorphous nanoparticles (Fig. 2b) and the distribution of distance between the centers of two amorphous nanoparticles (Fig. 2c). The average and standard deviation of the distribution of external radius for the amorphous nanoparticles in Kibushi Kaolin is 4.87(5) nm and 2.08(6) nm, respectively, where the values in parenthesis are estimated errors. The distribution of distance between two different amorphous nanoparticles is described with the scale of the occupancy of amorphous nanoparticles, where the occupancy is the volume ratio of amorphous nanoparticles in total space. The occupancy has a peak at the distance of about 8 nm and the occupancy decreases with increasing distance. This indicates that amorphous nanoparticles are aggregated and make secondary particles. The SAXS patterns, the distributions of external radius and the distribution of distance between the centers of two amorphous nanoparticles for all the samples are shown in Supplementary Figure S2. Table 3 shows the weight % of amorphous nanoparticles, the average external radius and the radius of void of amorphous nanoparticles for all the samples.

**Table 3:** Weight % of amorphous nanoparticles, volume distributions of external radius and void radius.

No	Amorphous nanoparticles/weight %	Volume distributions of external radius		Void radius/nm
		Average /nm	Standard deviation/nm	
571	19 (5)	6.9 (1)	3.6 (1)	1.59 (3)
572	5 (1)	6.6 (1)	3.8 (4)	1.72 (4)
573	10 (3)	5.9 (1)	3.3 (4)	1.68 (4)
574	16 (4)	1.5 (2)	1.0 (2)	0
575	25 (6)	4.0 (2)	1.8 (3)	1.67 (3)
576	8 (2)	4.2 (2)	1.9 (6)	1.69 (6)
577	10 (3)	5.5 (2)	3.3 (4)	1.62 (3)
578	32 (8)	3.00 (5)	0.69 (5)	1.63 (3)
579	82 (21)	3.31 (5)	1.17 (3)	1.56 (2)
806	36 (9)	4.61 (5)	1.83 (6)	1.38 (2)
807	42 (11)	4.87 (5)	2.08 (6)	1.38 (2)
808	26 (7)	4.95 (6)	2.24 (8)	1.41 (2)
809	19 (5)	4.66 (6)	2.00 (8)	1.35 (2)
861	75 (19)	5.65 (7)	3.2 (2)	1.56 (2))
862	25 (6)	3.07 (5)	0.79 (5)	1.55 (2)
863	47 (12)	4.16 (4)	1.5 (1)	1.44 (3)
864	15 (4)	3.6 (4)	1.3 (6)	1.77 (5)
867	21 (5)	2.81 (8)	0.80 (8)	1.60 (3)
868	10 (2)	3.36 (8)	0.9 (1)	1.64 (4)
869	35 (9)	2.95 (7)	0.85 (7)	1.64 (3)
870	21 (5)	2.4 (4)	0.7 (3)	1.55 (6)
871	26 (7)	3.2 (2)	0.8 (2)	1.85 (6)
872	73 (18)	5.07 (5)	2.40 (8)	1.47 (2)
873	26 (7)	3.04 (5)	0.81 (5)	1.52 (2)
874	47 (12)	3.09 (4)	0.89 (3)	1.42 (2)
875	47 (12)	3.12 (4)	0.89 (3)	1.43 (2)
876	10 (2)	3.20 (8)	1.0 (1)	1.56 (4)
877	36 (9)	2.9 (1)	1.0 (1)	1.64 (3)
878	18 (4)	3.9 (3)	2.5 (4)	1.60 (3)
879	25 (6)	4.2 (5)	2.7 (7)	1.74 (4)
901	39 (10)	3.69 (6)	1.28 (8)	1.49 (3)
902	43 (12)	3.63 (4)	1.26 (4)	1.38 (2)
903	18 (5)	2.67 (6)	0.73 (3)	1.13 (2)
904	28 (7)	4.21 (6)	1.6 (1)	1.59 (4)
905	27 (7)	4.15 (9)	1.2 (1)	1.33 (5)
906	37 (9)	3.17 (7)	0.76 (6)	1.60 (3)

Values in parentheses represent the estimated errors.

**Figure 3:**
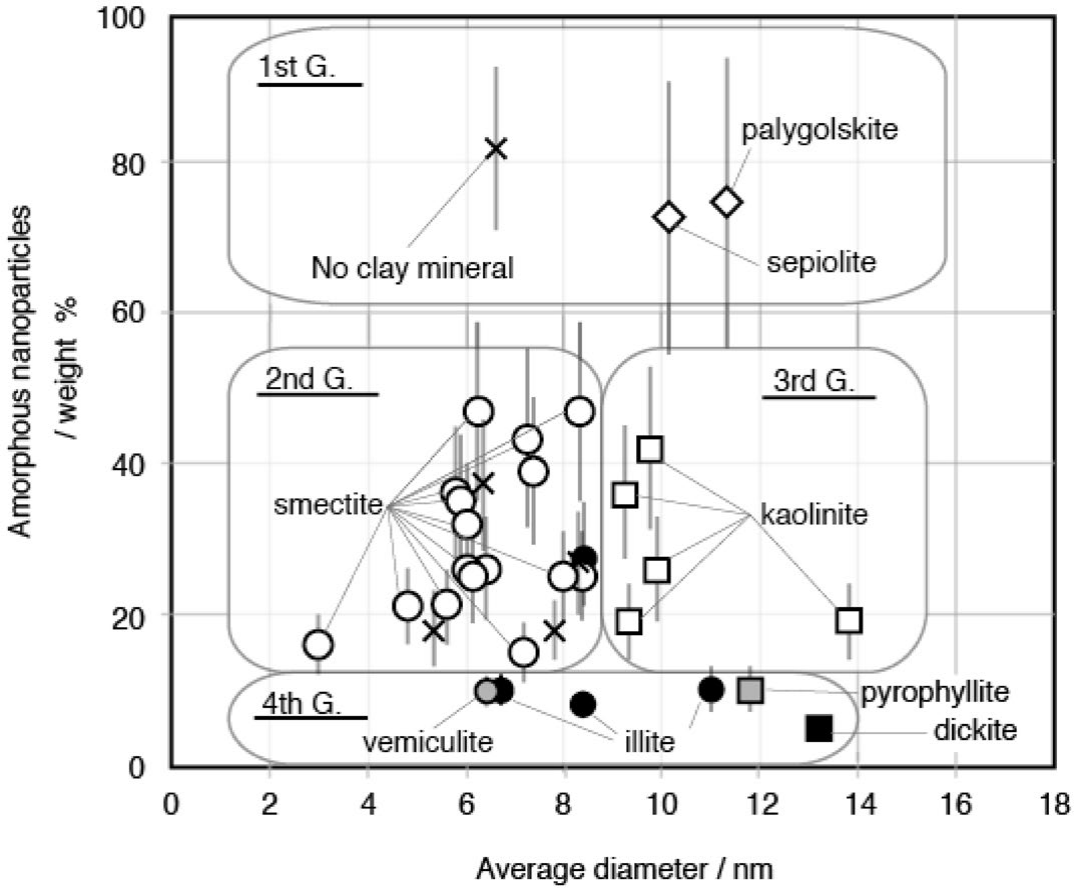
Weight % and average diameter of amorphous nanoparticles. These samples are classified into four groups according to the average diameter and the amount of amorphous nanoparticles.

On the basis of the average external diameter of amorphous nanoparticles and their weight %, the samples are divided into four groups (Fig. 3). First group is consists of samples containing amorphous nanoparticles in excess of 70 weight %. The group includes one soil with no clay mineral and two clays containing palygorskite or sepiolite. The second group is consists of samples containing 15–50 weight % of amorphous nanoparticles and with the average diameters of amorphous nanoparticles smaller than 9 nm. The group includes 17 clays containing smectite, one soil containing illite, three soils with no clay mineral, one marine-sediment containing smectite and one marine-sediment with no clay mineral. Note that all the samples containing smectite fall into this group. The third group is consists of samples containing 15 to 50 weight % of amorphous nanoparticles and with the average diameters of amorphous nanoparticles larger than 9 nm. The group includes five clays containing kaolinite. Note that all the samples containing kaolinite fall into this group. The fourth group is consists of samples containing 5–15 weight % of amorphous nanoparticles. The samples contain dickite, illite, pyrophyllite, or vermiculite. It is considered that these clay minerals in the fourth group were formed at high temperatures compared with smectite and kaolinite.

### Properties given by amorphous nanoparticles

**Figure 4:**
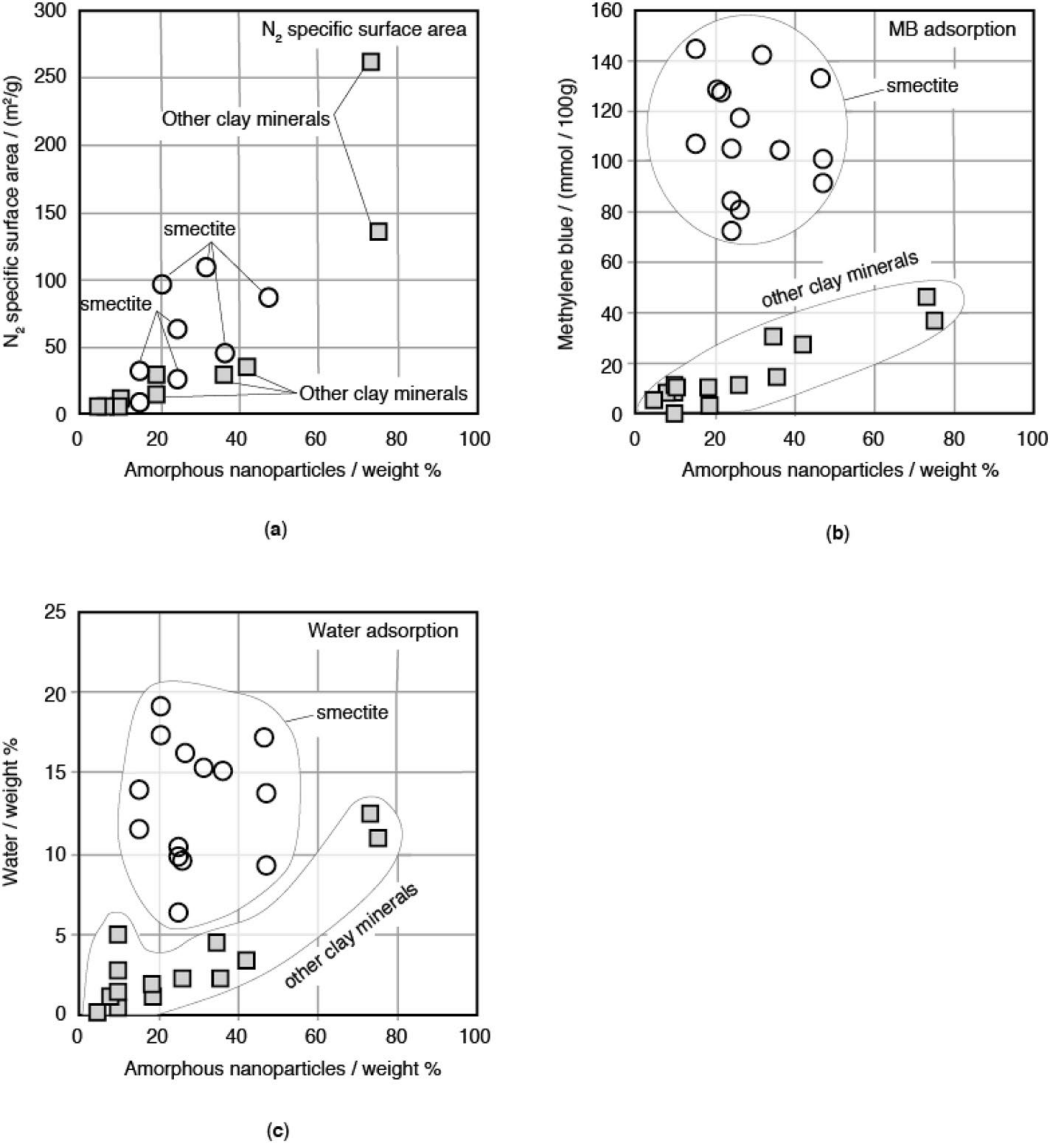
N_2_ specific surface area, and amounts of methylene blue and water absorbed on solids as a function of the weight % of amorphous nanoparticles. (**a**) N_2_ specific surface area. (**b**) Methylene blue adsorption. (**c**) Water adsorption.

Table 4 shows the N_2_ specific surface area, the amount of methylene blue adsorbed on solids (clay minerals and amorphous nanoparticles), which were measured with a colorimetric method^22^, and the amount of water adsorbed on solids. Figure 4a shows the N_2_ specific surface area as a function of the weight % of amorphous nanoparticles. The N_2_ specific surface area is directly proportional to the amounts of amorphous nanoparticles. This indicates that amorphous nanoparticles have large N_2_ specific surface area, and clay minerals have small N_2_ specific surface area. Figure 4a shows that the N_2_ specific surface areas of clays with smectite are slightly larger than the N_2_ specific surface areas of clays with kaolinite. This will be attributed to the sizes of amorphous nanoparticles; the amorphous nanoparticles in clays with smectite (2nd Group) are smaller than the amorphous nanoparticles in clays with kaolinite (3rd Group), which makes the surface areas of clays with smectite larger than the surface areas of clays with kaolinite.

**Table 4:** N_2_ specific surface area and concentration of methylene blue and water absorbed on solid.

No	N_2_ specific surface area /(m^2^/g)	Methylene blue/(mmol /100 g)	Water/weight %
571	14.82*	3.4	1.2
572	3.69*	5.4	0.2
573	4.63*	1.1	0.6
574	7.08*	144.6	13.9
575	27.35*	84.9	9.8
576	4.60*	8.2	1.2
577	10.21*	9.9	1.5
578	104.79*	142.1	15.4
579	212.74	19.3	15.1
806	30.42	14.5	2.3
807	33.22	26.7	3.4
808		10.8	2.3
809	24.52	9.9	1.9
861	136.35**	37.7	10.9
862	63.19**	72.5	6.4
863	83.79**	101.4	13.8
864	31.82**	107.8	11.5
867	97.42**	127.8	19.1
868		11.6	2.7
869		31.3	4.5
870		129.1	17.3
871		117.4	16.3
872	261.15	45.9	12.5
873		81.1	9.6
874	84.10	91.9	9.3
875		132.9	17.3
876		7.8	5.1
877	46.53	104.1	15.1
878	29.10	21.1	9.4
879		104.8	10.5

*Reference^19^.

**Reference^20^.

Figure 4b shows the amounts of methylene blue adsorbed on solids (clay minerals and amorphous nanoparticles) as a function of weight % of amorphous nanoparticles, which were measured with a colorimetric method^23^. When a sample contains smectite, the amount of methylene blue adsorbed on solids is large, and does not depend on the weight % of amorphous nanoparticles. This indicates that smectite adsorbs a large amount of methylene blue. When samples do not contain smectite, the amount of methylene blue adsorbed on solid is small to moderate, and the amount of methylene blue adsorbed on solids is directly proportional to the amounts of amorphous nanoparticles. This indicates that amorphous nanoparticles moderately adsorb methylene blue, and clay minerals other than smectite hardly adsorb methylene blue.

Figure 4c shows the amounts of water adsorbed on solids as a function of weight % of amorphous nanoparticles. When a sample contains smectite, the amount of water adsorbed on solid is large and does not depend on the weight % of amorphous nanoparticles. This indicates that the smectite adsorbs a large amount of water. When a sample contains a clay mineral other than smectite, the amount of water adsorbed on solids is directly proportional to the amounts of amorphous nanoparticles, and the sample with a large amount of amorphous nanoparticles adsorbs a large amount of water. This indicates that amorphous nanoparticles adsorb a large amount of water, whereas clay minerals other than smectite hardly adsorb water.

**Figure 5:**
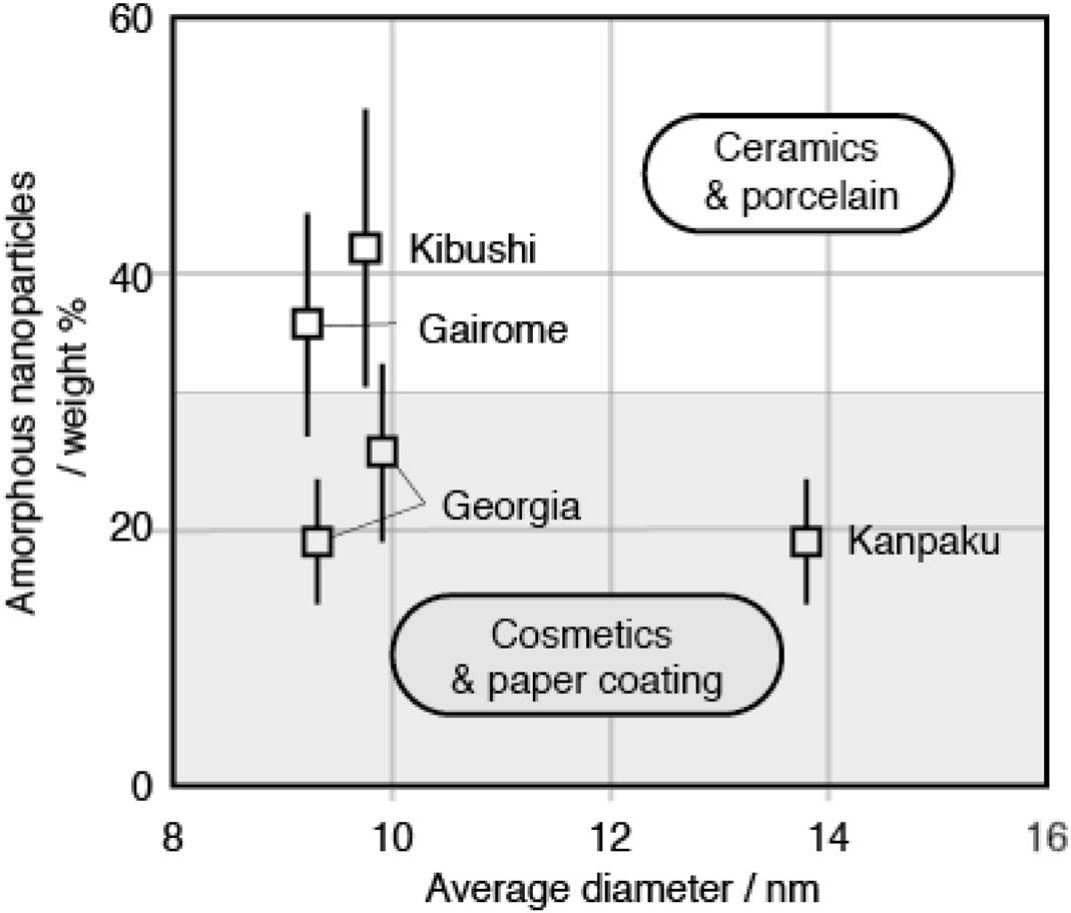
Weight ratio of amorphous nanoparticles in kaolin clays and their usage.

Amorphous nanoparticles in clays seem to increase the plasticity of the clays. Figure 5 shows the amounts of amorphous nanoparticles in two kinds of kaolin (clay containing kaolinite), which differ in the application. Kibushi kaolin and Gairome kaolin are used as raw materials for ceramics due to their high plasticity. On the other hand, Kanpaku kaolin and Georgia kaolin are not used for ceramics due to their low plasticity, and are used for cosmetics and paper coatings. Note that Kibushi kaolin and Gairome kaolin with higher plasticity contain more amorphous nanoparticles than Kanpaku kaolin and Georgia kaolin with lower plasticity.

## Discussion

We have various methods to analyze amorphous nanoparticles, such as small angle X-ray scattering (SAXS), transmission electron microscopy (TEM), photon correlation spectroscopy (PCS) and X-ray diffraction (XRD)^24,25^. Among them SAXS is the best method to analyze amorphous nanoparticles in clays, soils and marine sediments because SAXS can determine the size distribution of amorphous nanoparticles and quantify amorphous nanoparticles in a solid state. Although TEM can observe the shape and size of amorphous nanoparticles, TEM cannot determine the size distribution of amorphous nanoparticles and cannot quantify amorphous nanoparticles. Although PCS can determine the size distribution of amorphous nanoparticles in solution, PCS cannot determine the size distribution of amorphous nanoparticles in a solid state and cannot quantify amorphous nanoparticles. The inability of PCS to measure the size distribution of amorphous nanoparticles in the solid state is fatal because amorphous nanoparticles in clays, soils, and marine sediments are aggregated and dispersing all amorphous nanoparticles in solution is very difficult. Although XRD can determine the average size of crystalline materials, XRD cannot determine the size distribution of amorphous nanoparticles and cannot quantify amorphous nanoparticles.

We have to note that amorphous nanoparticles differ from volcanic glass. Although amorphous nanoparticles and volcanic glass are the same in that they are amorphous and have no reflection peaks in X-ray scattering pattern, amorphous nanoparticles differ from volcanic glass in two points. First, the formation mechanism differs between volcanic glass and amorphous nanoparticles. Volcanic glasses are formed by rapid cooling of molten rock formed at high temperatures (> 800 °C), while amorphous nanoparticles are formed by precipitation from aqueous solution at low temperatures (< 300 °C). At high temperatures, rocks melt and become amorphous because amorphous (high entropy) phases are thermodynamically stable at high temperatures. On the other hand, at low temperatures, amorphous nanoparticles precipitate fast and crystalline phases precipitate extremely slowly. Therefore, large amounts of amorphous nanoparticles are formed at low temperatures. Second, the sizes differ between volcanic glass and amorphous nanoparticles. Volcanic glass can be observed with an optical microscope^26^ because volcanic glass is usually larger than 1 μm. Their large sizes, however, produce weak intensities of SAXS. On the other hand, amorphous nanoparticles cannot be observed with an optical microscope because amorphous nanoparticles are too small (4 to 20 nm). Their small sizes, however, produce strong intensities of SAXS.

We have shown that materials formed at low temperatures (< 300 °C) on Earth such as clays, soils and marine sediments always contain large amounts of amorphous nanoparticles. Experimental studies showed that at low temperatures (< 300 °C) amorphous nanoparticles were precipitated first from a supersaturated solution because the formation rate of amorphous nanoparticles is fast compared with that of a crystalline phase, and that the amorphous nanoparticles were slowly transformed to a crystalline phase because the crystalline phase is more stable than amorphous nanoparticles^27–29^. Therefore, we consider that amorphous nanoparticles are formed first when rocks are weathered or altered in natural systems, and that the amorphous nanoparticles slowly transformed to a crystalline phase in the natural systems.

The rate of crystallization (transformation to a crystalline phase) of amorphous nanoparticles depends on the temperature and chemical composition of a system. Amorphous nanoparticles crystallize faster at high temperature than at low temperature. This is consistent with the result that a sample contains a small amount of amorphous nanoparticles (or a large amount of a crystalline phase) when the sample contains a crystalline phase formed at high temperatures (dickite, pyrophyllite or illite). The chemical composition also affects the rate of the crystallization of amorphous nanoparticles. The crystallization is fast when the chemical composition of the system is close to that of the crystalline phase, and the crystallization is slow when the chemical composition of the system is far from that of the crystalline phase^27^. The presence of impurities also retards the crystallization of amorphous nanoparticles^30^.

The diameter distributions of amorphous nanoparticles show that amorphous nanoparticles other than allophane seem to be present in clays, soils and marine sediments. The diameters of amorphous nanoparticles (4–16 nm in diameter) determined in this study (Supplementary Figure S2) are similar to or larger than those of allophane (3.5–5.8 nm in diameter^1,3^). Some of amorphous nanoparticles with diameters smaller than 5.8 nm in the samples may be allophane, but amorphous nanoparticles with diameter larger than 5.8 nm will be different kinds of amorphous nanoparticles other than allophane^2^. Amorphous nanoparticles with large diameters other than allophane seem to be present ubiquitously in clays, soils and marine sediments.

Most void diameters of amorphous nanoparticles measured in this study range from 2.8 to 3.4 nm. On the other hand, structural model of allophane showed that the void diameter of allophane is 4.0 nm^31^; this void diameter of allophane is somewhat larger than the void diameter of the amorphous nanoparticles measured in this study. This may be because clays, soils and marine sediments contain amorphous nanoparticles other than allophane or may be because the structure model of allophane differs from the real structure of allophane.

In this study we have measured the amount of methylene blue adsorbed on solids with a standard colorimetric method^22^. This method, however, may have underestimated the amount of methylene blue adsorbed on the amorphous nanoparticles because some amorphous nanoparticles may disperse and pass through a filter. In such case, some methylene blue adsorbed on the amorphous nanoparticles is not counted as adsorbed on solids. Therefore, we plan to clarify the amounts of amorphous nanoparticles that pass through the filter in future studies.

Amorphous nanoparticles adsorb many kinds of molecules and ions. In this study we have shown that amorphous nanoparticles adsorb a moderate amount of methylene blue and a large amount of water. Synthetic hydrous aluminum–silicate amorphous nanoparticles (HAS-clay) adsorb large amount of water and carbon dioxide^18^, and is attracting attention as an adsorbent for water and carbon dioxide. Amorphous nanoparticles also adsorb other kinds of molecules and ions, such as phosphate and sulfate^32^, organic matter^33,34^, phosphorus^35^ and arsenate^36^.

Methylene blue is adsorbed on solid surfaces by electrical attraction. Methylene blue becomes a cation in aqueous solution and is adsorbed on the negatively charged surface of solids (clays and amorphous nanoparticles)^23^. The layer of smectite is negatively charged and it is presumed that the surface of amorphous nanoparticles is also negatively charged. On the other hand, water enters the fine pores of solids, which are electrically neutral. Water adsorbed by smectite can be two different states: the water in the interlayer space and the water in external regions^37^. Water adsorbed by amorphous nanoparticles also can be two different states: the water in micro pores in the crust of amorphous nanoparticles and the water in the pores between amorphous nanoparticles^38^.

The radioactive cesium emitted from the Fukushima Daiichi nuclear power plant during the accident in 2011 will be adsorbed on amorphous nanoparticles in soils, but not much research has been done on the adsorption of cesium on amorphous nanoparticles. The SAXS intensities of the soils contaminated with radio-cesium in Fukushima area were strong^13–15^, which indicates that the soils contain large amounts of amorphous nanoparticles. The research on the adsorption of cesium, however, focused on clay minerals and ignored amorphous nanoparticles. Experimental study showed that weathered biotite adsorbed large amount of cesium^39^. High-angular annular dark-field imaging with scanning transmission electron microscopy and high-resolution electron microscopy showed that vermiculite and phlogopite incorporated cesium in interlayer sites^40–42^. It is certain that cesium is adsorbed on clay minerals, but it is highly probable that cesium is also adsorbed on amorphous nanoparticles.

In this study we have shown that kaolin containing a large amount of amorphous nanoparticles has a high degree of plasticity and kaolin containing only a small amount of amorphous nanoparticles has a low degree of plasticity. Amorphous nanoparticles seem to give the material plasticity, but kaolinite does not. Kaolinite should not be called a "clay mineral" if kaolinite does not give the material plasticity because "clay minerals" is minerals that give the material plasticity^43,44^. On the other hand, some researchers proposed that amorphous nanoparticles should be called clay minerals^45^ although amorphous nanoparticles are usually not called clay minerals because they are not crystalline.

## Methods

### X-ray scattering

Wide-angle scattering (WAXS) intensities were measured with a diffractometer equipped with diffracted beam monochromator with Cu *Kα* radiation at 40 kV and 100 mA. The scan rage is 2 to 40° 2θ, a step width is 0.02° 2θ and a scan rate is 0.5° 2θ per minute. Divergent slit is 1/6° and receiving slit is 0.3 mm.

Small angle X-ray scattering (SAXS) intensities were measured with a multipurpose X-ray diffractometer setting the optical system for SAXS measurement. The scan rage is 0.10 to 12.00° 2θ, a step width is 0.02° 2θ and a scan rate is 0.1° 2θ per minute. Raw SAXS intensities were converted to normalized SAXS intensities, where the normalized SAXS intensities are the scattering intensities per unit incident X-ray intensity, per unit weight of sample and without absorption^8^. These normalized intensities were calculated from raw intensities using the incident direct beam intensity, the transmitted direct beam intensity, and the mass absorption coefficient calculated from the chemical composition. A diffraction peak from a clay mineral and the background were subtracted from the normalized scattering intensities. The background was determined by averaging the intensities near 9° of 2θ. A diffraction peak of a clay mineral in less than 10.0° 2θ was approximated with one or two kinds of normal distribution curves.

By integrating the normalized SAXS intensities over 3-dimensional reciprocal space, we have obtained the value (the integral SAXS intensity) that is proportional to the weight % of amorphous nanoparticles^8^. To obtain the proportionality coefficient between the integral SAXS intensity and the weight % of amorphous nanoparticles, we measured SAXS intensities of colloidal silica (LUDOX SM30), whose weight % of amorphous nanoparticles was known. We have calculated the weight % of amorphous nanoparticles from the integral SAXS intensity and the proportionality coefficient.

By changing the distribution of the radius of amorphous nanoparticles, the distance distribution between the centers of two amorphous nanoparticles and the radius of the void, we have fitted the calculated SAXS intensities with the observed SAXS intensities. In this calculation, we applied the decoupling approximation^8,16^, and assumed that the distribution of the radius was a volume-logarithm normal-distribution, and a void was present in the center of amorphous nanoparticles. The procedure gives us the most probable distributions of radius of amorphous nanoparticles (Fig. 2b, Supplementary Figure S2), the most probable distribution of distance between centers of two nanoparticles (Fig. 2c, Supplementary Figure S2), and the most probable radius of the void (Table 3).

### Water and methylene blue adsorption

Samples was dried at 105–110 °C for > 18 h. We assumed that the weight % reduction in this procedure was attributed to the weight % of water adsorbed on solids. The dry sample (0.500 g) was placed in a conical beaker with 50 mL of 2% tetrasodium pyrophosphate solution. The solid grains in the solution were dispersed by ultrasonic treatment of 10 min. Methylene blue (MB) solution (0.01 mol/L) was added to the solution containing solid-grain dispersions. The mixed solution was stirred with magnetic stirrers for 24 h. Then, about 3 mL of solution was extracted from the solution with a 10-mL syringe. The extracted solution was filtered with a syringe filter that had a pore size of 0.2 μm (25AS020AN; advantec). The filtrates contained MB that was not adsorbed onto solids. The filtrates were diluted using propylene measuring flask. The MB concentration of the solution was measured with a spectrophotometer (U-5100; Hitachi, Tokyo, Japan) at a wavelength of 655 nm. The maximum amount of MB that could be added onto 100 g of a sample was determined by subtracting the amount of MB measured with the spectrometer from the amount of MB added to the solid dispersion.

## Supplementary Information

Supplementary Information
