# Supplementary material for: Amorphous nanoparticles in clays, soils and marine sediments analyzed with a small angle X-ray scattering (SAXS) method
Source: Sci Rep. 2021 Mar 26;11:6997. doi: 10.1038/s41598-021-86573-9 (PMC7997965; doi:10.1038/s41598-021-86573-9)
Supplement: Supplementary file 1 — Supplementary Information [file 41598_2021_86573_MOESM1_ESM.docx]

Amorphous nanoparticles in clays, soils and marine sediments analyzed with a small angle X-ray scattering (SAXS) method

Katsuhiro Tsukimura*^1^, Youko Miyoshi^1^, Tetsuich Takagi^1^, Masaya Suzuki^1^ & Shin-ichiro Wada^2^

^1^Institute for Geo-Resources and Environment, AIST (National Institute of Advanced Industrial Science and Technology), Tsukuba, Ibaraki 305-8567, Japan

^2^Faculty of Agriculture, Kyusyu University, Fukuoka, Fukuoka 819-0395, Japan

**Supplemetary Figure S1.** Wide-angle scattering data.

**Supplemetary Figure S2.** Distribution of radius of amorphous nanoparticles, distribution of distance between centers of two amorphous nanoparticles, and small angle X-ray scatterings (blue circles: observation, black dots: calculation) .

Dまれていた﷽﷽﷽﷽﷽﷽﷽﷽﷽﷽﷽
